# Dose–response relationship between intergenerational contact frequency and depressive symptoms amongst elderly Chinese parents: a cross-sectional study

**DOI:** 10.1186/s12877-020-01751-0

**Published:** 2020-09-15

**Authors:** Yaofei Xie, Mengdi Ma, Wenwen Wu, Yupeng Zhang, Yuting Zhang, Xiaodong Tan

**Affiliations:** 1 Wuhan University, No.115 of Donghu Road, Wuhan, 430000 China; 2 Wuhan Blood Center, No.8 of Baofeng One Road, Wuhan, 430000 China

**Keywords:** China, Depressive symptoms, Dose–response, Elderly, Intergeneration

## Abstract

**Background:**

Given the high prevalence of depressive symptoms amongst the elderly Chinese population and the significance of intergenerational contact in this demographic group, the purpose of this study was to examine the association and dose–response relationship between the frequency of intergenerational contact and depressive symptoms.

**Methods:**

Data were obtained from the third wave of the China Health and Retirement Longitudinal Study. A total of 5791 participants at age 60 or older were included in this study. Depressive symptoms were defined by the 10-item version of the Centre for Epidemiologic Studies Depression Scale. Intergenerational contact included in-person meeting and remote connecting, and they were analysed separately. Intergenerational contact frequency was classified into ten categories and then treated as a continuous variable for analysis. We performed univariate and multivariate logistic regressions to identify risk covariables. Restrictive cubic spline analysis was used to examine the dose–response relationship between intergenerational contact frequency and the outcome of depressive symptoms.

**Results:**

Both the frequency of meeting and the frequency of connecting with children were independently associated with depressive symptoms in the elderly, and the odds ratios for depressive symptoms increased with decreasing frequencies (*P* < 0.01). There was a negative dose–response relationship between intergenerational contact frequency and depressive symptoms. The odds of depressive symptoms steadily decreased with increasing frequency of meeting with their children. Following an initial increase, the odds rapidly decreased as the frequency of connecting with children increased with an inflection point at once a monthly. Both associations were nonlinear (*P* < 0.001).

**Conclusions:**

Our findings revealed a negative dose–response relationship between intergenerational contact frequency and depressive symptoms in the elderly Chinese population. Thus, future health interventions should consider cultural norms in shaping the mental well-being of Chinese elderly persons.

## Background

Depressive disorders amongst the elderly population is a worldwide public health problem [1]. The Global Health Estimates reported by the World Health Organization (WHO) indicate that the prevalence of depressive disorders has peaked amongst the elderly [2]. In contrast to depressive disorders in younger populations, late life depression (major depressive disorders) is a risk factor for many chronic medical conditions [3, 4]. In addition, depression can result in significantly lower quality of life and increase mortality in the elderly population [5–7].

Depressive symptoms are the major manifestations of depressive disorders. The prevalence of depressive symptoms varies between regions and populations. A study of elderly Latinos in the United States reported a 25.5% prevalence of depressive symptoms [8]. Another study reported a 14.0% prevalence in elderly Americans of Chinese descent [9]. Two population-based studies conducted in Germany [10] and Greece [11] reported 28.7 and 32.3% prevalence of depressive symptoms amongst elderly, respectively. About 19.0% of elderly Koreans had depressive symptoms [12]. The 2008 Chinese Elderly Mental Health Survey revealed that over 39% of the elderly participants self-reported depressive symptoms [13]. The 2011 China Health and Retirement Longitudinal Study (CHARLS) also reported that approximately 40% of older adults had depressive symptoms [14]. China is facing a rapidly aging society; the proportion of elderly has grown from 13.3% [15] to 17.9% [16] of the population between 2010 and 2018. Thus, there is a corresponding growth in the number of older people displaying depressive symptoms.

Previous studies have found that contact frequency with offspring is inversely associated with depressive symptoms amongst older parents [17–19]. One explanation is that older parents expect support from their children, and when their adult children do not meet expectations, depressive symptoms may arise [20]. It has been suggested that intergenerational contact is a critical aspect of social support for the elderly [21], and the lack of which has been implicated in an increased risk of depressive symptoms [22]. Indeed, intergenerational contact provides an opportunity for companionship, sharing of interests and opinions, and for expression of emotions. For the elderly, these opportunities foster a sense of belonging to the family, and they also enable family members to offer help and care [23]. Children may provide a sense of meaning and order to life, and they may even be a source of self-esteem and prestige for the elderly [24].

Intergenerational contact is important for elderly Chinese people. Chinese Confucian cultures emphasises family and filial piety, and the practice of intergenerational contact is a part of filial piety. The absence of filial piety in the lives of Chinese elderly is connected with depressive symptoms [25–27]. One study in elderly Chinese-American also reported that those experiencing greater than expected filial piety had a lower risk of depressive symptoms [28].

More than ever, elderly Chinese people experience less contact with their adult children. According to the China Health and Retirement Report published by the CHARLS team in 2019, the number of elderly people not living in the same city as any of their children rose from 8 to 11% between 2011 and 2015. In the same period, there was also an increase from 19 to 24% in adult children living in different cities from their parents, and increasing distance between children and parents was associated with less intergenerational contact [29]. In addition, the proportion of ‘empty nesters’ (i.e., parents who do not live with their adult children) have increased remarkably and now includes nearly half of the elderly population in the country [30]. It is estimated that the proportion of empty-nester households could reach 90% by 2030 [31].

Several studies reported a high prevalence of depressive symptoms amongst empty nesters [32–34]. However, to our knowledge, no study has investigated the direct association between intergenerational contact frequency and depressive symptoms in the elderly Chinese population. To address this gap in knowledge, the present study in elderly Chinese participants aims to: (a) examine the association between intergenerational contact frequency and depressive symptoms, and (b) explore its dose–response relationship.

## Methods

### Samples

Data were obtained from the 2015 third wave survey data from the CHARLS study. CHARLS is an ongoing longitudinal study that collects demographic, social and economic data as well as the health status of nationally representative samples of middle-aged and elderly Chinese residents. CHARLS uses a four-stage sampling method. At the county and village levels, a probability- proportional- to- size sampling method was used, and a total of 450 villages or communities were included. At the household and individual levels, the CHARLS researchers designed a software package named CHARLS-Geographic Information System (CHARLS-GIS) to determine each sampling frame on the map. Furthermore, a sample of 80 households was randomly selected from a list of all households in each sampling frame. In every household sample, a family member over 45 years old was randomly selected as the main interviewee. Face-to-face interviews were then conducted by trained investigators to collect study data. The response rate was over 85% [35]. All the survey data are publicly available online. A total of 5791 participants were retained in the present study after removing the participants younger than 60 years and the participants with missing values on any of the main variables. The sample size was considered sufficient.

### Depressive symptoms

Depressive symptoms were determined using the 10-item version of the Centre for Epidemiologic Studies Depression Scale (CES-D-10) [36]. The Cronbach’s alpha of the Chinese version of this scale was 0.813 [37].

The CES-D-10 contains three items addressing depressive affect, five addressing somatic symptoms, and two specifying positive affect. Each item has four options: option 1, “Rarely or none of the time (< 1 day)”, option 2, “Some or a little of the time (1 − 2 days)”, option 3, “Occasionally or a moderate amount of the time (3 − 4 days)”, option 4, “Most or all of the time (5 − 7 days)”. Participants are asked to recall the frequency of specific feelings and behaviours during the past week and to choose the most appropriate response. Each item was rated from 0 to 3, corresponding to the first through fourth options. Exception include ‘Item 5’ and ‘Item 8’, which are reverse-scored from 3 to 0. The total score of the CES-D-10 scale ranged from 0 to 30, and participants who scored ≥10 points were considered to have significant depressive symptoms [38].

### Intergenerational contact

Two intergenerational contact types were analysed: in-person “meetings” and remote “connections” made either by phone, text message, postal mail or email. The frequency was categorised into ten numbered items, 1 = almost never, 2 = less than once a year, 3 = once a year, 4 = once every 6 months, 5 = once every 3 months, 6 = once a month, 7 = every 2 weeks, 8 = once a week, 9 = 2–3 times a week and 10 = almost every day. The participants were allowed to select the number closest to the actual frequency. The contact frequency of all adult children was recorded and co-resident children were included under the category of “almost every day”.

### Covariates

We collected basic demographic characteristics including gender, age, marital status, education level, and residence that have been reported to be associated with depressive symptoms amongst the elderly Chinese population as control variables [39–41]. Previous studies reported that the presence of chronic disease was significantly related to depressive symptoms in the elderly [42, 43]. Conversely, old parents in poor physical health may stimulate increased contact with their children. So we used the variable of “number of chronic diseases suffered” to reduce the possibility of reverse causation. We analysed the intergenerational contact frequencies of all children in a family using the variable of “number of children” as a covariate to control for the effect of having multiple children.

### Statistical analysis

We first conducted a descriptive analysis of all the study variables. Pearson chi-square tests and variance analyses were performed to assess differences in CES-D-10 scores and the prevalence of depressive symptoms in the subgroups. Next, univariate and multivariate logistic regression models were used to calculate the unadjusted and adjusted odds ratios (ORs) of the covariates to identify risk factors for depressive symptoms. Finally, we used restrictive cubic splines with four knots at the 25th, 50th, 75th, and 95th centiles to explore the dose–response relationship between intergenerational contact frequency and depressive symptoms. In the restrictive cubic spline analysis, the significant risk factors identified earlier were adjusted in the model. The frequency of “meetings” and the frequency of “connections” were mutually adjusted. A likelihood ratio was used to test for non-linearity [44, 45]. Missing values were accounted by using regression interpolation. Statistical significance was set to *P* < 0.05, and all *P-*values were two-sided.

## Results

### Participants and depressive symptoms

Table 1 displays the demographic characteristics, CES-D-10 scores, and the prevalence of depressive symptoms. Among the 5791 participants, males accounted for about 56%, the average age was 69 years old, and over 60% were from 60 to 69 years old. Nearly two-thirds of the participants were living with their spouse, and more than 70% lived in villages. Uneducated participants accounted for about 30% of the sample, and nearly half of them completed education no further than elementary-level. Nearly 60% of their household consumption expenditures per capita fell within 0–2000 RMB. More than 80% of participants reported that they were diagnosed with at least one type of chronic disease. Over 90% of participants had more than one child.

In terms of intergeneration contact frequency, participants that met with their children either at least once a week or 1–2 times a month accounted for approximately 30% of the sample each. Over a quarter of participants met with their children 1–2 times every 6 months, and about 12% reported a frequency of ≤1 time per year. The participants who connected with their children either ≥2 times or 1 time a week accounted for approximately one-third of the sample each. About 15% of participants connected with their children 1–2 times a month, while others reported the frequency at ≤1 time every 3 months.

The mean CES-D-10 score of all the participants was 8.83 ± 6.68. Using ≥10 as the cut-off score, depressive symptoms were identified in 37.52% of the participants. The variance analysis and chi-square test results revealed that gender, marital status, residence, education level, number of chronic diseases, number of children, frequency of meeting with children, and frequency of connecting with children were each associated with differences in CES-D-10 scores and in the prevalence of depressive symptoms (*P* < 0.001).

### Depressive symptoms and associated factors

Table 2 presents the results of the logistic regression models for the associated factors of depressive symptoms. The unadjusted ORs for significant variables were calculated. We tested for collinearity on all independent variables using tolerance and variance inflation factors (VIFs) before multivariate analyses. All values for tolerance were over 0.1 and all VIFs were less than 10.0, therefore, there was no collinearity among independent variables.

**Table 1:** Participant characteristics by CES-D-10 scores and depressive symptoms

Variables	N (%)	CES-D-10 scores (Mean ± SD)	***F***	Depressive symptoms (N / prevalence %)	***χ***^**2**^
**Gender**			209.159^***^		157.471^***^
Male	3216 (55.53)	7.72 ± 6.14		977 (30.38)	
Female	2575 (44.47)	10.23 ± 7.05		1196 (46.45)	
**Age (years old)**			1.138		2.646
60–69	3504 (60.51)	8.74 ± 6.70		1318 (37.61)	
70–79	1791 (30.93)	9.03 ± 6.70		685 (38.25)	
≥ 80	496 (8.57)	8.73 ± 6.48		170 (34.27)	
**Marriage status**			64.053^***^		83.448^***^
Living with spouse present	3788 (65.41)	8.12 ± 6.36		1262 (33.32)	
Married but not living with spouse temporarily	190 (3.28)	9.42 ± 6.48		81 (42.63)	
Divorced / Widowed / Never married	1813 (31.31)	10.25 ± 7.11		830 (48.78)	
**Residence**			52.735^***^		102.656^***^
Main city zone	916 (15.82)	6.62 ± 5.82		226 (24.67)	
Town area	540 (9.32)	7.80 ± 6.38		167 (30.93)	
Township	255 (4.40)	8.42 ± 6.28		88 (34.51)	
Village	4080 (70.45)	9.49 ± 6.80		1692 (41.47)	
**Education level**			75.525^***^		190.701^***^
Illiterate	1705 (29.44)	10.57 ± 7.18		812 (47.62)	
Elementary school or below	2706 (46.73)	8.91 ± 6.56		1030 (38.06)	
Middle school	851 (14.70)	6.92 ± 5.75		229 (26.91)	
High school or Vocational school	410 (7.08)	6.02 ± 5.44		81 (19.76)	
Bachelor’s degree / Associate degree or greater	119 (2.05)	5.53 ± 4.65		21 (17.65)	
**Household per capita consumptions expenditure last year**^a^			0.936		3.474
≤ 2000 RMB / 286.69 US$ / 255.90 €	3302 (57.02)	8.87 ± 6.71		1233 (37.34)	
~ 5000 RMB / 716.72 US$ / 639.75 €	1233 (21.29)	9.00 ± 6.47		487 (39.50)	
~ 10,000 RMB / 1433.44 US$ / 1279.51 €	630 (10.88)	8.57 ± 6.98		223 (35.40)	
> 10,000 RMB / 1433.44 US$ / 1279.51 €	626 (10.81)	8.58 ± 6.62		230 (36.74)	
**Number of chronic diseases suffered**^b^			135.991^***^		246.78^***^
None	1103 (19.05)	6.57 ± 5.52		264 (23.93)	
One	1452 (25.07)	7.83 ± 6.22		468 (32.23)	
Two	1309 (22.60)	8.52 ± 6.37		466 (35.60)	
Three or more	1927 (33.28)	11.09 ± 7.15		975 (50.60)	
**Number of children**			25.595^***^		11.394^**^
One	498 (8.60)	7.39 ± 6.20		152 (30.52)	
Two or more	5293 (91.40)	8.97 ± 6.71		2021 (38.18)	
**Frequency of meeting with children**			35.732^***^		79.056^***^
At least once a week	1725 (29.79)	7.70 ± 6.37		524 (30.38)	
1–2 times a month	1793 (30.96)	8.66 ± 6.44		653 (36.42)	
1–2 times every 6 months	1533 (26.47)	9.64 ± 6.82		656 (42.79)	
Once a year or less	740 (12.78)	10.22 ± 7.17		340 (45.95)	
**Frequency of connecting with children**			28.873^***^		60.018^***^
At least twice a week	1985 (34.28)	7.80 ± 6.32		611 (30.78)	
Once a week	1948 (33.64)	8.99 ± 6.61		782 (40.14)	
1–2 times a month	900 (15.54)	9.85 ± 7.01		375 (41.67)	
Once every 3 months or less	958 (16.54)	9.70 ± 6.93		405 (42.28)	
**Total**	5791 (100.00)	8.83 ± 6.68		2173 (37.52)	

^*^P < 0.05; ^**^P < 0.01; ^***^P < 0.001

^a^ The exchange rates of RMB against US dollar and euro were the rates on January 1, 2020. The absolute poverty line of Chinese population in 2019 was 2300 RMB annual per capita disposable income

^b^A total of 14 types of chronic diseases were asked: 1. Hypertension; 2. Dyslipidemia (elevation of low density lipoprotein, triglycerides (TGs), and total cholesterol, or a low high density lipoprotein level); 3. Diabetes or high blood sugar; 4. Cancer or malignant tumor (excluding minor skin cancers); 5. Chronic lung diseases, such as chronic bronchitis, emphysema (excluding tumors, or cancer); 6. Liver disease (except fatty liver, tumors, and cancer); 7. Heart attack, coronary heart disease, angina, congestive heart failure, or other heart problems; 8. Stroke; 9. Kidney disease (except for tumor or cancer); 10. Stomach or other digestive disease (except for tumor or cancer); 11. Emotional, nervous, or psychiatric problems; 12. Memory-related disease; 13. Arthritis or rheumatism; 14. Asthma

**Table 2:** Multivariable logistic regression of factors associated with depressive symptoms

Independent Variable	Unadjusted OR (95% CI)	***P***	Adjusted OR ^a^ (95% CI)	***P***
**Gender (Male as reference)**				
Female	1.99 (1.78–2.21)	< 0.001	1.69 (1.49–1.92)	< 0.001
**Marriage Status (Living** **with spouse present** **as reference)**				
Married but not living with spouse temporarily	1.49 (1.11–2.00)	0.008	1.36 (1.00–1.85)	0.054
Divorced / Widowed / Never married / Cohabitated	1.69 (1.51–1.89)	< 0.001	1.29 (1.13–1.47)	< 0.001
**Residence** **(Main city zone** **as reference)**				
Town area	1.37 (1.08–1.73)	0.010	1.20 (0.93–1.54)	0.155
Township	1.61 (1.19–2.17)	0.002	1.35 (0.98–1.86)	0.066
Village	2.16 (1.84–2.55)	< 0.001	1.78 (1.47–2.14)	< 0.001
**Education level (Illiterate as reference)**				
Elementary school or below	0.68 (0.60–0.76)	< 0.001	0.88 (0.76–1.01)	0.060
Middle school	0.40 (0.34–0.48)	< 0.001	0.64 (0.52–0.78)	< 0.001
High school or Vocational school	0.27 (0.24–0.35)	< 0.001	0.46 (0.35–0.63)	< 0.001
Bachelor’s degree / Associate degree or greater	0.24 (0.15–0.38)	< 0.001	0.48 (0.29–0.80)	0.005
**Number of chronic diseases suffered (None as reference)**				
One	1.51 (1.27–1.80)	< 0.001	1.44 (1.20–1.73)	< 0.001
Two	1.76 (1.47–2.10)	< 0.001	1.74 (1.44–2.09)	< 0.001
Three or more	3.25 (2.76–3.84)	< 0.001	3.34 (2.81–3.96)	< 0.001
**Number of children (One as reference)**				
Two or more	1.41 (1.15–1.71)	0.001	0.96 (0.77–1.19)	0.702
**Frequency of meeting with children** **(Over once a week** **as reference)**				
1–2 times a month	1.31 (1.14–1.51)	< 0.001	1.20 (1.04–1.40)	0.016
1–2 times every 6 months	1.71 (1.48–1.98)	< 0.001	1.49 (1.28–1.74)	< 0.001
Once a year or less	1.95 (1.63–2.33)	< 0.001	1.68 (1.39–2.03)	< 0.001
**Frequency of connecting with children (At least twice a week as reference)**				
Once a week	1.51 (1.32–1.72)	< 0.001	1.28 (1.07–1.52)	0.006
1–2 times a month	1.61 (1.36–1.89)	< 0.001	1.32 (1.11–1.58)	0.002
Once every 3 months or less	1.65 (1.40–1.93)	< 0.001	1.34 (1.17–1.55)	< 0.001

^a^ adjusted for gender, marriage status, residence, education level, number of chronic diseases suffered, number of children, frequency of meeting with children, frequency of connecting with children.

*OR* odds ratio and *CI* confidence intervals

Adjusted regression analysis showed that depressive symptoms were more likely to be identified in women (OR: 1.69, 95% CI: 1.49–1.92); those who were divorced, widowed, never married, or cohabitated (OR: 1.29, 95% CI: 1.13–1.47); those who lived in villages (OR: 1.78, 95% CI: 1.47–2.14); those with a low education level; those who met up with their children less than once weekly; and those who connected with their children less than twice weekly. The odds of depressive symptoms increased with an increasing number of chronic diseases: 44, 74, and 234% for one, two, and three or more conditions, respectively. There was no significant association between the number of children and depressive symptoms.

### Depressive symptoms and intergenerational contact

**Fig. 1:**
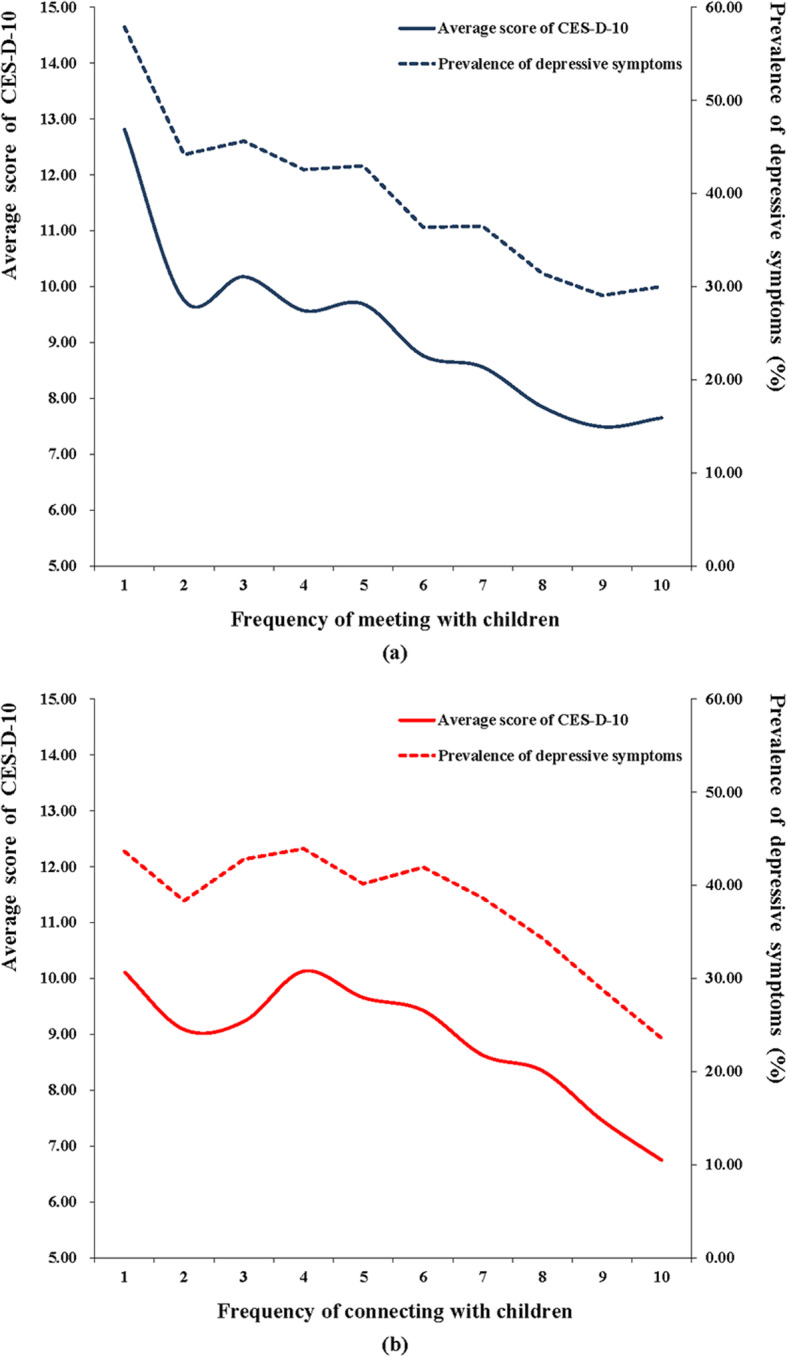
Changes in CES-D-10 scores and in the prevalence of depressive symptoms relative to intergenerational contact frequency. Frequency: 1 = almost never, 2 = less than once a year, 3 = once a year, 4 = once every 6 months, 5 = once every 3 months, 6 = once a month, 7 = every 2 weeks, 8 = once a week, 9 = 2–3 times a week and 10 = almost every day

Multiple logistic regression results demonstrated that intergenerational contacts were independently associated with depressive symptoms amongst the elderly Chinese population. Figure 1 illustrates the changes in CES-D-10 scores and prevalence of depressive symptoms in conjunction with (a) meeting with children in-person and (b) connecting with children remotely through different methods. Overall, it shows that lower CES-D-10 scores and fewer depressive symptoms tend to trend with increasing frequency of intergenerational contact.

**Fig. 2:**
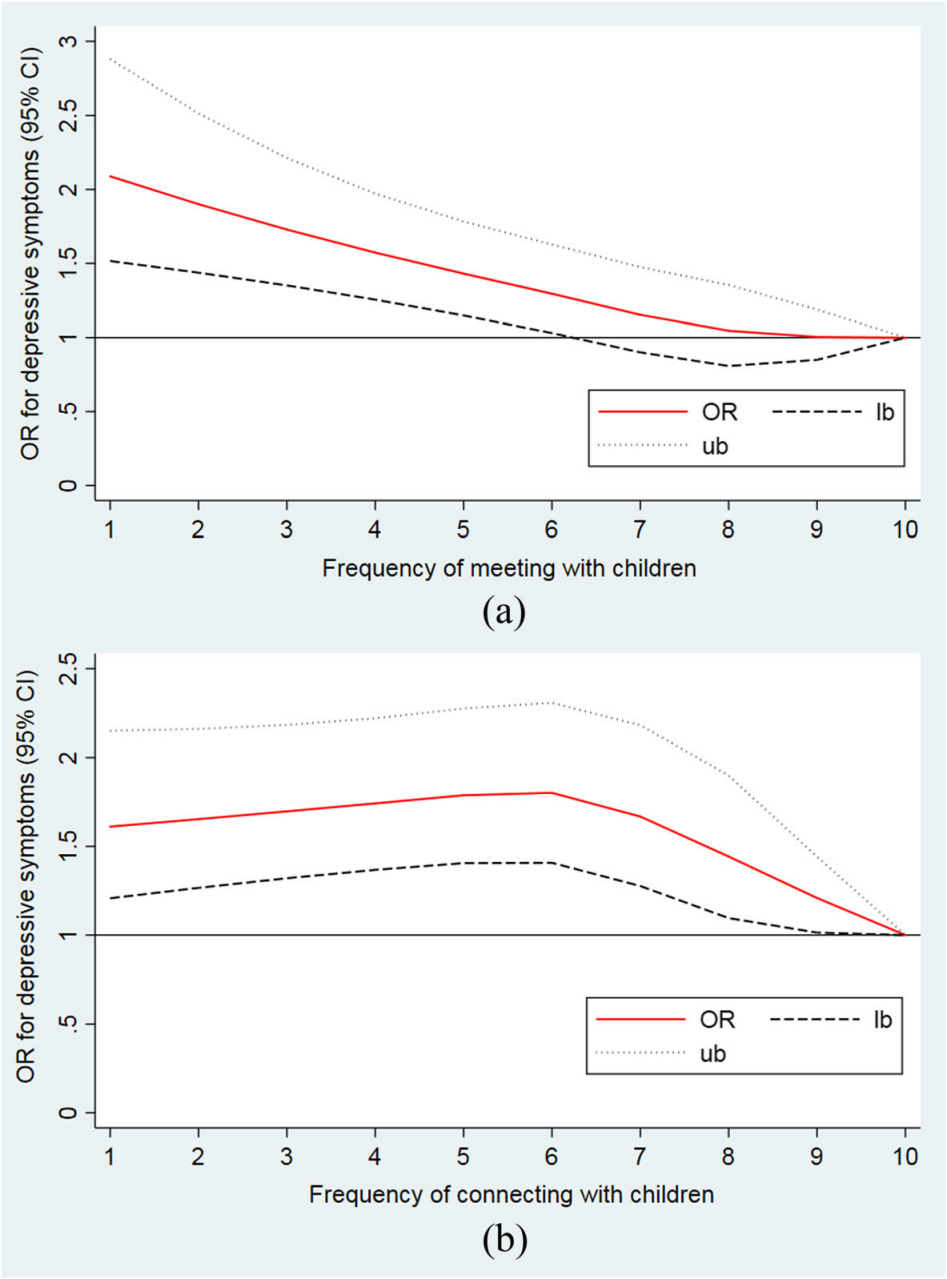
Dose–response association between intergenerational contact frequency and the odds for depressive symptoms. Frequency: 1 = almost never, 2 = less than once a year, 3 = once a year, 4 = once every 6 months, 5 = once every 3 months, 6 = once a month, 7 = every 2 weeks, 8 = once a week, 9 = 2–3 times a week and 10 = almost every day. OR: odd raio; lb.: lower limit; ub: upper limit

Figure 2 shows the dose–response relationship between depressive symptoms and intergenerational contact frequency resulting from restricted cubic splines analyses. The analyses were adjusted for the factors associated with depressive symptoms in multivariable logistic regression. Both models were nonlinear (*P* < 0.001 for the non-linearity test).

Figure 2(a )illustrates the steadily decreasing ORs for depressive symptoms with an increasing frequency of meeting with children. Remarkably, the odds of depressive symptoms show a significant inverse association with contact frequency when it falls below once monthly (Frequency of meeting with children = 6). Meanwhile, Fig. 2(b) shows the ORs for depressive symptoms increased slightly followed by a sharp decrease as the frequency of connecting with children increases. The inflection point for the changes in ORs occurs at once-monthly connections. (Frequency of connecting with children = 6).

## Discussion

The increasing social–structural constraints on resources and time have reduced intergenerational contact between the elderly Chinese population and their adult children. Considering China’s traditional family culture and the special importance of intergenerational contact for this group, the present study investigated the relationship between intergenerational contact and depressive symptoms.

We found overall prevalence of depressive symptoms in the participants was 37.5% as defined by a CES-D-10 score of ≥10, which is higher than the prevalence reported in the elderly Japanese population of 11.5%. That study defined depressive symptoms using the Geriatric Depression Scale (GDS) with a cut-off score of ≥6 [46]. Our result is also higher than the prevalence of 31.5% reported in elderly Europeans; the results there were measured using the EURO-D scale with a cut-off score of ≥4 [47]. By contrast, the prevalence for institutionalised elderly persons in Brazil was 45.8% [48] and it was 60.6% in elderly Nepalese [49]. Depressive symptoms in these studies were also defined by the GDS with a cut-off score of ≥6. The differences in these findings may be attributed to the economic and cultural differences amongst different regions.

Depressive symptoms amongst the elderly Chinese are associated with low levels of education, unmarried status, and location setting of residence, which are consistent with several previous studies [39, 40, 50].

Amongst the elderly Chinese, intergenerational contact frequency is independently and inversely associated with depressive symptoms; it exhibits a negative dose–response relationship with the odds of depressive symptoms. For in-person interactions, the ORs for depressive symptoms did not increase significantly with a decrease of frequency of interaction until they reached a frequency of ≤1 time monthly compared with those who have almost daily contact. The results suggest that children should meet up with their older parents at least once a month to possibly prevent them from developing depressive symptoms. This result is similar to a study in the U.S. that reported that the frequency of in-person interactions with children was inversely associated with the development of depressive symptoms in older parents [51]. For contact by telephone, postal mail, or email, relative to connecting almost everyday, the ORs for depressive symptoms rapidly increased with a decrease in the frequency of connection. They reached an inflection point where connections were made once a month, whereby the ORs decreased slightly with a decrease in contact frequency. We suggest that other forms of contact are more easily accessible, therefore parents expect a greater frequency of interaction with their children, and they develop depressive feelings more easily when there is a minor decrease in contact frequency. But when the frequency drops to once monthly, their depressive feelings reach a stable level but do not get worse. During the coronavirus disease 2019 (COVID-19) pandemic, quarantines were implemented to prevent disease transmission, but concurrently, they had a negative psychological impact [52–54]. Our findings suggest that children should keep close connections with their older parents during periods of quarantine, which may help reduce the risk of depressive symptoms.

The associations of depressive symptoms and intergenerational contact frequency might be related to three aspects. First, infrequent intergenerational contact leads to loneliness in older adults, thereby resulting in increased depressive symptoms. Loneliness is a subjective and negative feeling that occurs when individuals experience diminished social relationships [55]. On the one hand, infrequent contact with family can heighten the risk for loneliness [56], because loneliness is usually conceptualized as perceived social isolation [57], and infrequent social contact is an objective measure of perceived social isolation [58]. Moreover, Chinese family culture attaches great importance to the happiness of a family union. Thus, the elderly Chinese are prone to loneliness when they are unable to receive sufficient companionship from their adult children. On the other hand, depressive symptoms were regarded as a logical consequence of loneliness [59]. Previous studies have shown that loneliness is consistently and strongly associated with depressive symptoms [60–62]. Therefore, loneliness may be an intermediate step between reduced intergenerational contact and depressive symptoms.

Second, infrequent intergenerational contact limits the availability of family support for the elderly. Social support for many elderly adults comes from family members. Specifically, intergenerational support is a primary component that may improve the psychological well-being of elderly parents [63]. Poulin et al. [64] argued that family support from adult children can prevent depressive symptoms in elderly Chinese people. Conversely, loss of support from children is a key reason for the high prevalence of depressive symptoms in this population [13]. Furthermore, part of intergenerational contact is the provision of home care, and it has been reported that the Chinese elderly who worry about the lack of caregivers tend to have higher levels of depressive symptoms [65]. Hence, decreased intergenerational contact may be related to reduced caregiving, thereby leading to increased depressive symptoms.

Third, infrequent intergenerational contact is not conducive to maintaining a good parent–child relationship. Poor relationships are associated with depressive symptoms in the elderly. Intergenerational contact is considered a critical indicator of the strength of the parent–child relationship [19]. Frequent contact provides opportunities for companionship and socialising, thus promoting the parent–child relationship. Li et al. [66] reported that parent–child relationships may directly affect depressive symptoms amongst the elderly Chinese population. In addition, a study in elderly people living in the rural areas of the U.S. similarly concluded that parent–child relationships were inversely associated with depressive symptoms [67].

Several limitations of this study should be addressed. We attempted to consider all children of survey participants who had multiple children. However, having infrequent contact with one child may matter less if other children maintain relatively more frequent contact. Also the effects of contact frequency on depressive symptoms may be different between each child. Thus, our use of the average frequency of intergenerational contact for parents with multiple children may produce bias. Investigating these potential effects is beyond the scope of our study, but it is important for future studies to investigate each parent–child relationship or factors that contribute to differences between each child. Next, we did not have study variables that described qualities stemming from intergenerational contact, such as loneliness, social support and parent–child relationships. Therefore, comprehensive and structural relationships for these variables were not obtained. Finally, the data were cross-sectional, which allowed us to analyse associations between variables, but a causal relationship between intergenerational contact and depressive symptoms could not be determined. To address these issues, future longitudinal analyses that feature additional variables should be conducted to enhance our understanding of the relationship between intergenerational contact and depressive symptoms.

## Conclusions

The present study demonstrates that lower intergenerational contact frequency with children is independently associated with greater depressive symptoms amongst the elderly Chinese population. The ORs for depressive symptoms show an increasing trend with decreasing frequency of contact with children of the participants. Our results suggest that provision of depression prevention services should be considered for older Chinese people. A number of demographic characteristics are related to depressive symptoms and should be taken into account during screening. Scholars and policy makers should pay attention to family cultural norms when health interventions are considered in the elderly Chinese population.

## Data Availability

The datasets analysed during the current study are available in the CHARLS repository: http://charls.pku.edu.cn/index/en.html.

## Abbreviations

CHARLS: China Health and Retirement Longitudinal Study
CHARLS-GIS: CHARLS-Geographic Information System
CES-D-10: The 10-item version of the Centre for Epidemiologic Studies Depression Scale
OR: Odds ratio
95% CI: The 95% confidence intervals
GDS: Geriatric Depression Scale
COVID-19: The coronavirus disease 2019
